# Common sequence variants in CD36 gene and the levels of triglyceride and high-density lipoprotein cholesterol among ethnic Chinese in Taiwan

**DOI:** 10.1186/1476-511X-11-174

**Published:** 2012-12-18

**Authors:** Kuo-Liong Chien, Hsiu-Ching Hsu, Pi-Hua Liu, Hung-Ju Lin, Ming-Fong Chen

**Affiliations:** 1 Institute of Epidemiology & Preventive Medicine, National Taiwan University, Taipei, Taiwan; 2 Department of Internal Medicine, National Taiwan University Hospital, Taipei 100, Taiwan; 3 Clinical Informatics and Medical Statistics Research Center, Chang Gung University, Tao-Yuan, Taiwan

**Keywords:** Metabolic syndrome, CD 36 gene polymorphism

## Abstract

**Background:**

Evidence of the genetic association between *CD36* candidate gene and the risk of metabolic syndrome and its components has been inconsistent. This case–control study assessed the haplotype-tagged SNPs from *CD36* on the risk of metabolic syndrome and components.

**Methods and results:**

We recruited 1,000 cases and age, gender-matched controls were randomly selected from the participants with metabolic syndrome defined by International Diabetes Federation. Overall, the haplotype tagged SNPs of CD36 gene were not related to the risk of metabolic syndrome. For individuals with normal lipid levels, several SNPs were significantly associated with the triglycerides and HDL-cholesterol levels: Subjects with rs3211848 homozygote had a higher triglyceride level (99.16 ± 2.61 mg/dL), compared with non-carriers (89.27 ± 1.45 mg/dL, *P* = 0.001). In addition, compared with non-carriers, individuals with rs1054516 heterozygous and homozygous genotypes had a significantly lower HDL-cholesterol (46.6 ± 0.46 mg/dL for non-carrier, 44.6 ± 0.36 mg/dL for heterozygous, and 44.3 ± 0.56 mg/dL for homozygous, *P* = 0.0008).

**Conclusion:**

The *CD36* gene variants were significantly associated with triglycerides and HDL-cholesterol concentrations among ethnic Chinese in Taiwan.

## Introduction

The CD36 is a glycoprotein located in membrane and plays various cellular processes such as lipid transport, immune regulation, coagulation and atherosclerosis [1], and the CD36 structure is related to scavenger receptor B1, and highly binds to oxidized LDL [2], which induced atherosclerosis process. *CD36* gene variants regulated fatty acid metabolism and accumulation of fat and fat metabolites, and may influence the risk of metabolic syndrome and may be a target for further personalized medicine screening [3].

Emerging evidence indicating that variation in the *CD36* gene may play a role in the pathogenesis of metabolic syndrome. Individuals with CD36 deficiency, which is prevalent among Asians, were likely to have the abnormal levels of triglycerides and HDL-cholesterol [4,5]. In addition, genetic variants of *CD36* were related to acute myocardial infarction [6], type 2 diabetes [7,8], metabolic syndrome components [9,10], fatty acids [9] and adiponectin levels [11], and free fatty acids [12]. However, no special ethnic Chinese population was reported and only modest effects have been identified for variants of *CD36* gene for metabolic syndrome, and the associations for metabolic syndrome itself have often been inconsistent. These inconsistent findings may be attributed to inadequate information from *CD36* gene. In addition, the confounding effects by clinical and lifestyle factors should be considered concurrently [13]. Therefore, we examined the association of common variants of the *CD36* gene with the metabolic syndrome and components using a case–control study among ethnic Chinese adults, controlling for clinical and lifestyle factors.

## Methods

### Study design and study participants

The study design was a case–control study design based on 8,911 adult participants who were recruited from the Health Management Center of one tertiary hospital from January 2004 to December 2007 and all provided the written informed consents with the study protocol being reported elsewhere [14,15]. In brief, details of socioeconomic status, along with medical and medication histories were collected by questionnaires, and standardized clinical procedures were undertaken. We excluded the participants with concurrent severe medical diseases such as cancer and heart failure. The participants signed informed consent forms, and the protocol was approved by the Institutional Research Board of the National Taiwan University Hospital.

Details of subjects’ medical histories such as medication, hospitalization and smoking status were included in the structural questionnaires. Standardized procedures for the physical examination, such as anthropometric measures and blood pressure, were performed [16,17]. Blood pressure was measured in a resting position by standard procedure. Body mass index (BMI) and standing height were measured with subjects dressed in light clothing and barefoot. BMI was calculated as weight (in kilograms)/square of height (in meters). Waist circumference was measured midline between the low costal margin and superior posterior iliac crest.

### Laboratory measurements

Procedures for blood sampling and analytic methods were as previously described [18,19]. In brief, a blood sample was collected from each participant after at least a 12 hours fasting. Serum total cholesterol levels were measured using the CHOD-PAP method (Boehringer Mannheim, Germany). HDL-C was measured following precipitation of apolipoprotein B-containing lipoproteins with phosphotungstic acid and magnesium ions (Boehringer Mannheim, Germany). Triglyceride concentrations were measured by the GPO-DAOS method (Wako Co., Japan). All of the lipids mentioned above were measured using a Hitachi 7450 automated analyzer (Hitachi, Japan). LDL-C concentrations were calculated using the Friedewald formula. All the measures of both samples were carried out in a single hospital and the coefficients of variation were less than 5%.

### Case ascertainment and matched control selection

We have recruited the participants from the matched case–control design during 2003 and 2007. We randomly selecting from 8,911 participants recruited from the Health Management Center and all participants provided written informed consent. We used International Diabetes Federation (IDF) criterion to define the metabolic syndrome cases. The IDF criterion included central obesity and other two components including high blood pressure, dyslipidemia and high glucose levels which were defined as: blood pressure of at least 130/85 mmHg or undergoing treatment for hypertension (39%); serum triglyceride of at least 150 mg/dL (25%); HDL cholesterol of <40 mg/dL in men and <50 mg/dL in women (53%); fasting glucose of 100 mg/dL or more (20%); and central obesity, waist circumference greater than 90 cm in men and 80 cm in women (38%). A total of 1000 cases of metabolic syndrome were randomly selected. In addition, the 1:1 matched control subjects were selected in the same samples, with frequency-matched according to age and gender.

### DNA genotyping & polymorphism detection

Three primers were designed for each SNP detection with web-based software provided at Beckman Coulter Inc (http://www.autoprimer.com). A forward and areverse PCR primers were used to amplify a short stretch of DNA (90 bp) covering the SNP of interest, and a tagged SNP primer for single base primer extension to identify the SNP. The 5' portion of the tagged primer is complementary to one of 12 unique single stranded DNA oligonucleotides that are spotted at a specific location within each well of a 384-well microplate. The 3' portion of the tagged primer is complementary and precisely adjacent to the SNP, which enables detection of the presence of the SNP through the incorporation of a fluorescent-labeled terminating nucleotide. The genotyping were performed in a 12-plex PCR reaction of a 384-well plate with the GenomeLab SNPstream genotyping platform (Beckman Coulter Inc. Fullerton, CA) and its accompanying SNPstream software suite. The PCR reaction was carried out in a total volume of 5 μl containing 75 μM dNTPs (Applied Biosystem), 5 mM MgCl_2_, 50 nM each primer, 2 ng of template DNA, and 0.1 U/μL Taq Gold (Applied Biosystem). The cycling conditions (MJ-225 thermal cycler) were 94°C for 1 minute followed by 39 cycles of 94°C for 30 seconds, 55°C for 30 seconds, and 72°C for 1 minute. After PCR reaction, the amplified DNA fragments were treated with a clean-up cocktail of exonuclease I and shrimp alkaline phosphatase to degrade unincorporated PCR primers and deoxynucleotide triphosphates. The reactions were carried out by adding 3 μl of a mixture containing 0.67 U Exonuclease I (Amersham Pharmacia, Buckinghamshire, UK) and 0.33 U shrimp alkaline phosphatase (Amersham Pharmacia) to each well. The plates were sealed and incubated for 30 min at 37°C and at 95°C for 10 min. The tagged extension reaction was carried out with ‘cleaned’ PCR products as template and a mixture of 12 site-specific SNP primers. The multiplex primer SNP extension reaction was performed in a total volume of 7 μl containing 3.76 μl SNP extension dilation buffer, 0.06 μl SNP primer mix (each 5 μM), 0.2 μl C/T (or C/G, T/C et al.) extension mix, 2.96 μl water, and 0.02 μl SNPware DNA polymerase. The cycling conditions were 96°C for 3 minutes followed by 45 cycles of 94°C for 20 seconds, and 40°C for 11 seconds. The tagged extension primers were extended with single TAMRA- or fluorescein-labeled nucleotide and then spatially resolved by hybridization to the complementary oligonucleotides arrayed on the 384-well microplates (SNPware Tag array). The Tag array plates were imaged with a two-laser, two-color charged couple device-based imager (GenomeLab SNPstream array imager). The 12 individual SNPs were identified by their position and fluorescent color in each well according to the position of the tagged oligonucleotides. Sample genotype data were generated on the basis of the relative fluorescent intensities for each SNP. The image signals were then transferred to genotyping software that translated the images of the arrays into genotype calls. The error rate on the basis of blind replicates was 0.1 to 1% for the SNPs examined in the present study.

Using the common SNPs for Han Chinese were identified from the International HapMap Project (http://www.hapmap.org), and we selected the tagged SNP for *CD36* candidate gene, which contained 19 SNPs whose minor allele frequency was more than 5%. Tagger program was used to select haplotype-tagging SNPs (htSNPs) based on the pairwise linkage disequilibrium relationships (r^2^ = 0.8). The common SNP subset represented the information from the remaining SNPs and was in linkage disequilibrium within each block.

### Statistical analysis

All data were presented as mean and standard deviation for continuous variables and contingency tables for categorical data and were listed by status of case patients and control subjects. The chi-square tests were used to access Hardy-Weinberg equilibrium among cases and controls and the allele frequency was compared between the cases of metabolic syndrome and control subjects. Subgroup analysis based on gender and age was performed for specific genotype. Relationships between metabolic component quantitative variables were analyzed by multiple linear regression analysis adjusting for age, gender in specific subjects within the normal ranges of the metabolic syndrome cutoff values. False discovery rate [20] were used to correct for the multiple comparison problems for the significant *P* value threshold as 0.05. In addition, we investigated the association between the genotypes of SNP rs1054516 and HDL cholesterol, after controlling clinical and lifestyle factors in the multiple regression model.

The selected tagged SNPs spanning *CD36* form one “block” whose algorithm was developed by Gabriel and colleagues[21], where blocks identified with the default settings in the Haploview program were merged if they have multiallelic D’ greater than 0.8 and the cumulative frequency of common haplotypes (>5% frequency) in the merged block is >80% [22]. Haplotype frequencies were estimated by SAS/Genetics software using the expectation-maximization algorithm and the results were verified by the THESIAS program [23]. Pair-wise linkage disequilibrium coefficients, Lewontin's *D*' and correlation coefficients (r^2^) were estimated iteratively. Haplotype-specific analyses assessing the association between haplotypes and the continuous lipid profiles, were performed by THESIAS program, which is based on the stochastic-estimation and maximization algorithm. The likelihood ratio test for haplotype-phenotype association was twice the difference between the log-likelihood under the null (one haplotype) and the alternative (additional haplotypes) model, and the chi-square with haplotype numbers as degree of freedom. We examined the global significance level in the multivariate adjusted linear models, and specifically tested the haplotype effects, compared with the most common haplotype for reference.

Previous literature showed that the relative risk between *CD36* SNP and the risk of metabolic syndrome was 1.3 ~ 1.4, and the allele frequencies of SNPs were 0.10 to 0.25 [9-11]. Under the additive mode of inheritance and the baseline risk for the metabolic syndrome as 0.10, we estimated the power for genetic effect was 90% with the sample size of 1000 cases & 1000 matched controls [24].

## Results

### Basic distribution of case–control subjects

Excluding missing data and failed genotyping, a total of 962 cases and 938 controls provided full genotyping data. The clinical distributions of clinical and metabolic components among the study participants, specified by metabolic syndrome are listed in Table 1. Compared with control subjects, case patients were likely to have the history of hypoglycemic and lipid lowering drug, a higher smoking and lower sports activity habit. In addition, compared with control subjects, case patients were likely to have a higher BMI, waist, blood pressure, fasting and postprandial glucose, glycated hemoglobin, uric acid levels and a higher total and LDL cholesterol, triglycerides, and lower HDL cholesterol levels.

**Table 1:** Clinical characteristics of the study participants according to the metabolic syndrome status

	**Control**	**%**	**Case**	**%**	**P**
	**N = 938**		**N = 962**		
	**N**		**N**		
Gender					0.60
Women	343	36.6	363	37.7	
Men	595	63.4	599	62.3	
Hypoglycemic drugs (Yes)	49	5.2	130	13.5	<.0001
Lipid lowering drugs (yes)	44	4.7	99	10.3	<.0001
Current smoker	132	14.1	173	18.0	0.020
Current drinker	539	57.5	545	56.7	0.72
Regular sports habit	669	71.3	636	66.1	0.014
	**Mean**	**SD**	**Mean**	**SD**	
Age, yrs	54.5	10.6	55.1	10.6	0.22
Body mass index, kg/m^2^	22.7	2.3	27.1	3.0	<.0001
Waist circumference, cm	79.2	6.4	94.2	7.1	<.0001
Systolic blood pressure, mmHg	122.7	14.8	133.2	14.9	<.0001
Diastolic blood pressure, mmHg	72.9	9.8	79.2	10.3	<.0001
Total cholesterol, mg/dL	203.0	36.5	206.8	36.7	0.025
Triglycerides, mg/dL	115.0	74.3	177.6	103.8	<.0001
HDL cholesterol, mg/dL	43.2	9.8	38.5	7.0	<.0001
LDL cholesterol, mg/dL	116.4	31.6	124.7	32.7	<.0001
Fasting glucose, mg/dL	93.3	22.7	105.2	28.1	<.0001
Postprandial glucose, mg/dL	120.1	52.3	129.8	59.2	0.0002
Uric acid, mg/dL	6.1	1.5	6.6	1.6	<.0001

### Association of *CD 36* gene variants and metabolic syndrome status

A total of 19 common htSNPs of *CD36* gene were selected from the HapMap website (http://www.hapmap.org) to further genotyping (Additional file 1: Table S1). We randomly selected 200 cases/200 controls from the study participants and genotyped these SNPs. From the pilot genotyping results, we found that several *CD36* gene SNPs were associated with metabolic syndrome status and metabolic trait, including HDL, triglycerides, body mass index, fasting glucose and triglyceride levels (Additional file 1: Table S2). Finally, we selected the 7 SNPs for completing genotyping work in all subjects.

Table 2 shows the minor allele frequency in the selected 7 SNPs for *CD36* gene in the study participants, call rates, and the Hardy-Weinberg equilibrium testing for cases and controls. We found that the call rates were satisfactory (>99%) and the Hardy-Weinberg equilibrium was not rejected in all SNPs, implying the genotyping quality was good. We cannot identify any SNP for a significant association with the status of metabolic syndrome for all subjects and limited to < 55 years old, as well as gender, in any mode of inheritance, including co-dominant, additive, dominant and recessive modes (data not shown).

**Table 2:** **Allele frequency in the selected SNPs for the *CD36 *gene (Chromosome #7) in the study participants and the Hardy-Weinberg equilibrium testing**

**rs#**	**Allele**	**Position**	**MAF**	**Controls**	**Call rate**	**HWE P value**	
			**Cases**			**Cases**	**Controls**
rs3211849	A < G	80121259	0.350	0.368	99.6%	0.68	0.08
rs17154258	G < A	80134866	0.055	0.056	99.8%	0.76	0.53
rs3211869	A < T	80125088	0.288	0.287	99.7%	0.48	0.15
rs3211958	G < A	80142008	0.442	0.421	99.7%	0.51	1.00
rs3211817	G < T	80116043	0.351	0.348	99.6%	1.00	0.39
rs1054516	C < T	80122878	0.447	0.455	99.8%	0.70	0.23
Rs17154233	C < A	80131125	0.134	0.119	99.8%	0.42	0.64

*MAF*: minor allele frequency.

**Figure 1:**
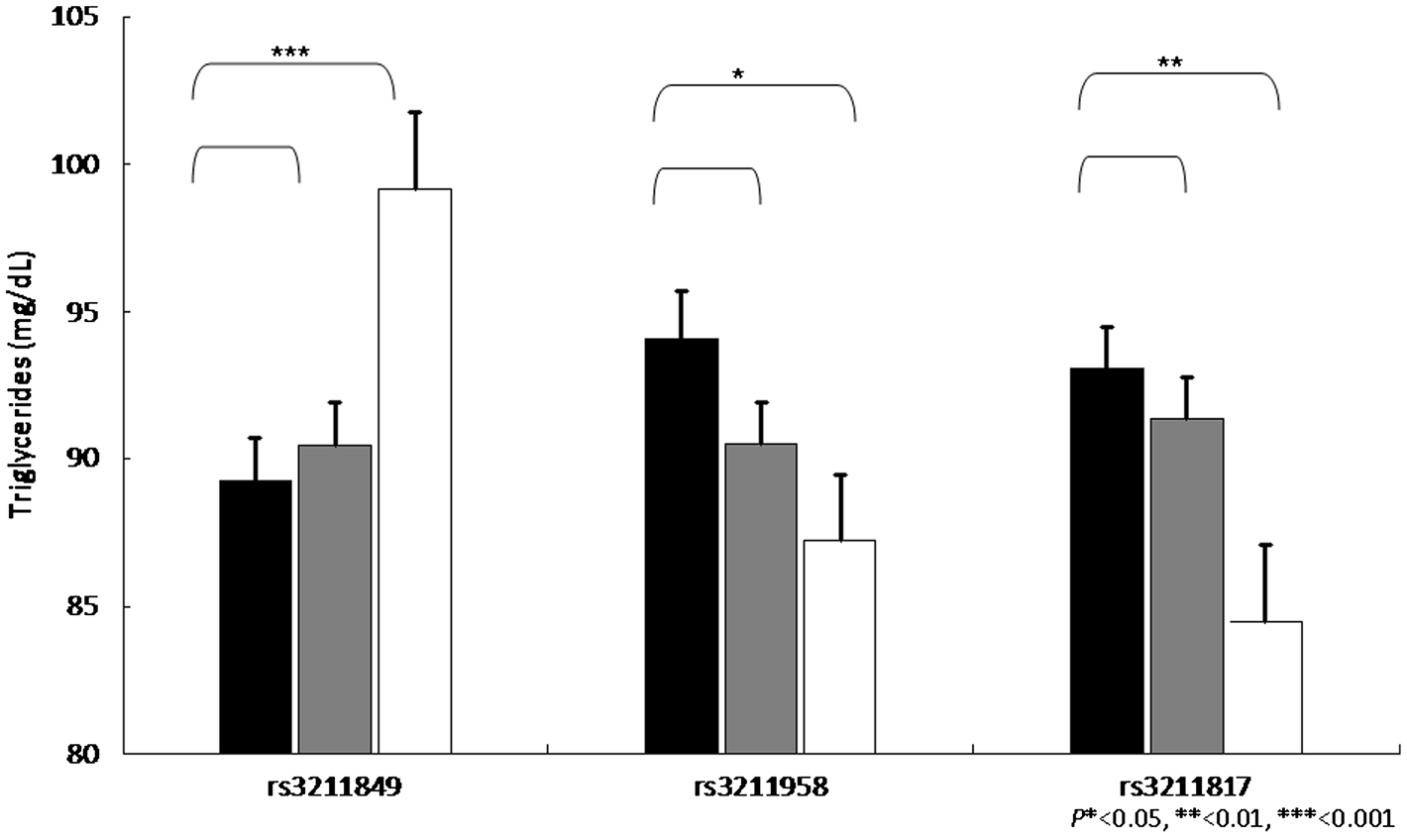
**Impact of CD36 gene on triglycerides levels (mg/dL ± SE) from pairwise genotype comparisons.** Adjusted mean triglycerides for non-carriers (black bars), for heterozygous (gray bars) and subjects homozygous for the minor allele (white bars). Sample sizes (n for non-carriers, heterozygous, and homozygous subjects for SNP (rs3211849), n = 383, 408, 119, for SNP (rs3211958), n = 305, 432, 174, and for SNPs (rs3211817) n = 377, 416, 115, resulting co-dominant P-values as 0.004, 0.035, and 0.018.

**Figure 2:**
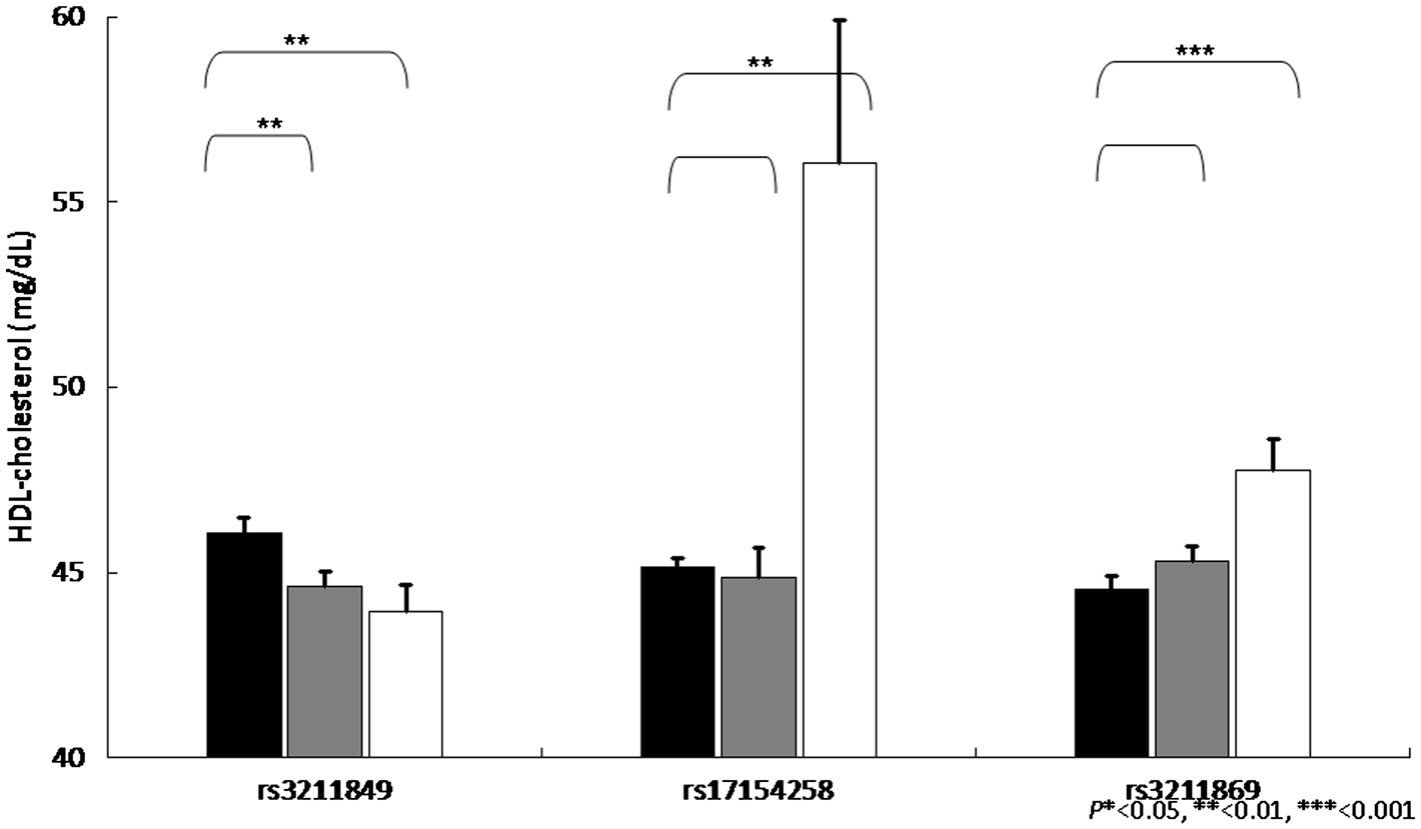
**Impact of *CD36 *gene on HDL-cholesterol levels (mg/dL ± SE) from pairwise genotype comparisons.** Adjusted mean HDL-cholesterol for non-carriers (black bars), for heterozygous (gray bars) and subjects homozygous for the minor allele (white bars). Sample sizes (*n* for non-carriers, heterozygous, and homozygous subjects for SNP (rs3211849), n = 383, 408, 119, for SNP (rs17154258),n = 813, 94, 4, and for SNPs (rs3211869), n = 463, 363, 84, resulting co-dominant *P*-values as 0.006, 0.017 and 0.002.

**Figure 3:**
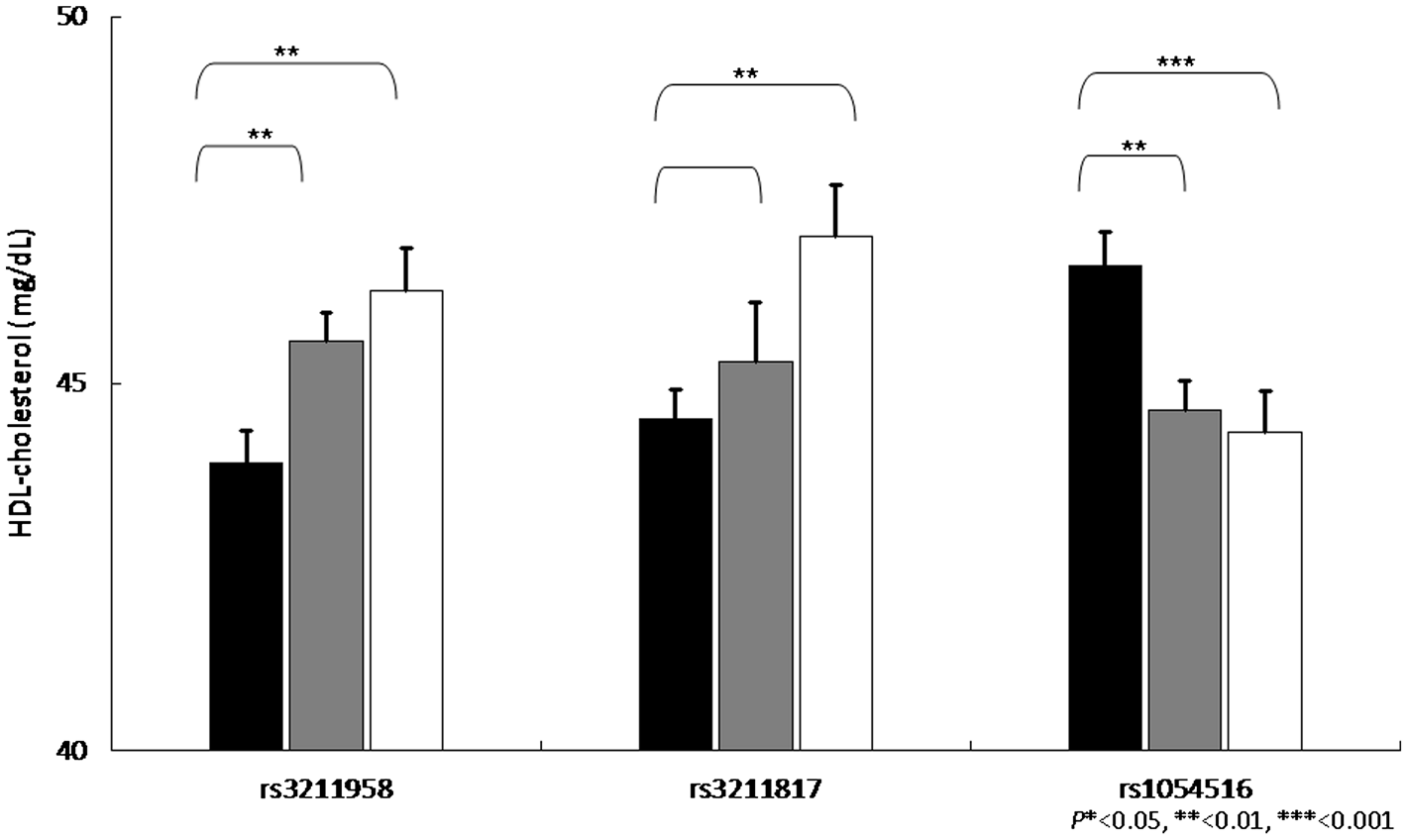
**Impact of *CD36 *gene on HDL-cholesterol levels (mg/dL ± SE) from pairwise genotype comparisons.** Adjusted mean HDL-cholesterol for non-carriers (black bars), for heterozygous (gray bars) and subjects homozygous for the minor allele (white bars). Sample sizes (*n* for non-carriers, heterozygous, and homozygous subjects for SNP (rs3211958), n = 305, 432, 174, for SNP (rs3211817), n = 377, 416, 115, and for SNP (rs1054516), n = 281, 444, 187, resulting co-dominant *P*-values as 0.002, 0.010, and 0.0008.

**Figure 4:**
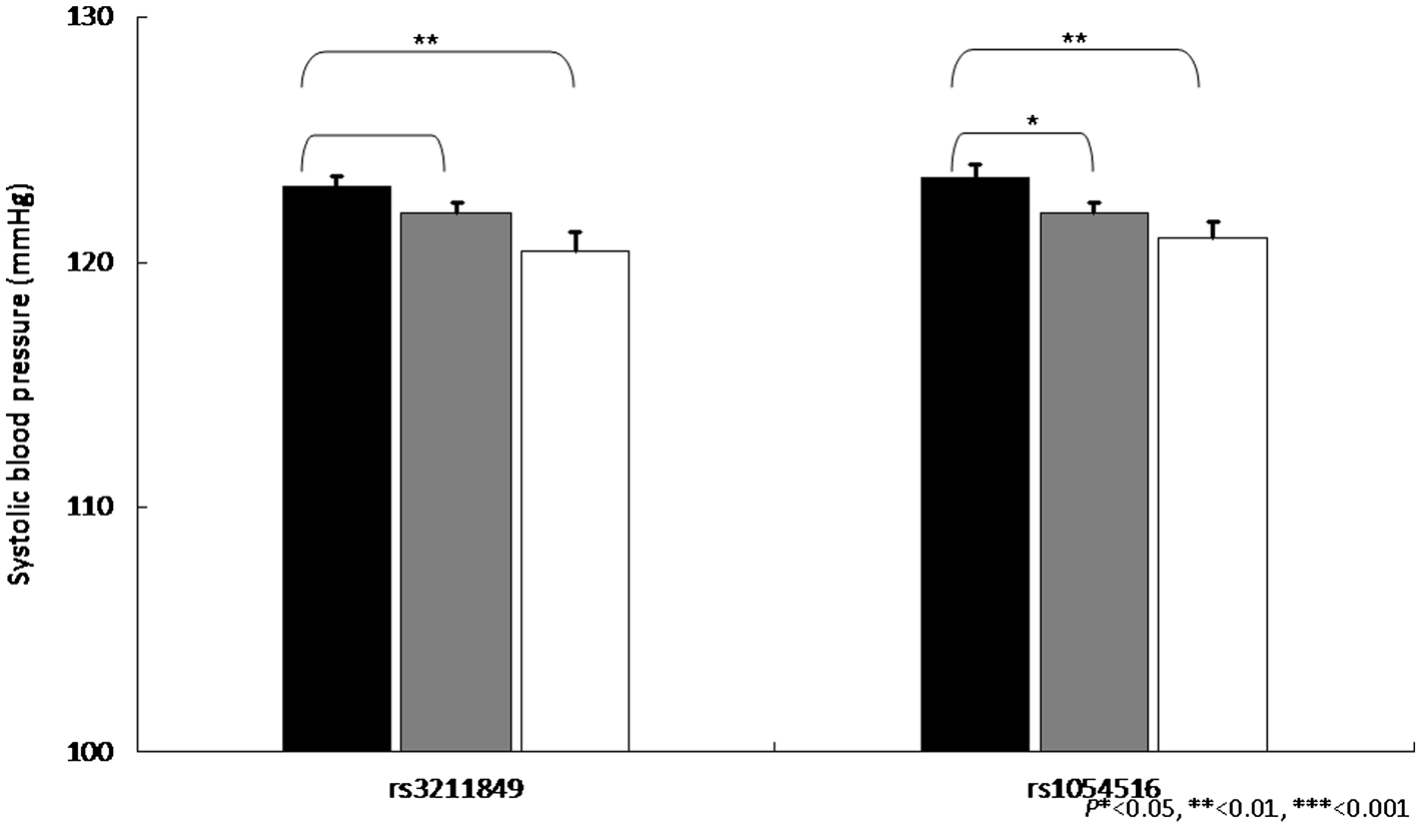
**Impact of *CD36 *gene on systolic blood pressure (mmHg ± SE) from pairwise genotype comparisons.** Adjusted mean systolic blood pressure for non-carriers (black bars), for heterozygous (gray bars) and subjects homozygous for the minor allele (white bars). Sample sizes (*n* for non-carriers, heterozygous, and homozygous subjects for SNP (rs3211849), n = 621, 664, 207, and for SNP (rs1054516), n = 466, 720, 308, Resulting co-dominant *P*-values as 0.009 and 0.007.

Our next strategy is to perform the genetic association between *CD36* gene SNPs and various metabolic syndrome components. Overall, the association between SNPs and metabolic components were found for triglycerides, HDL-cholesterol and blood pressure, as well as postprandial glucose, glycated hemoglobin and c-reactive protein. Among them, we found that triglycerides, HDL-cholesterol, triglycerides vs. HDL-cholesterol ratio, have the most significant level, and the significant level was between rs1054516 and HDL-cholesterol, with *P* < 0.001 in additive, dominant and co-dominant inheritance of models. Other SNPs, including rs3211849, rs17154258, rs3211869, rs3211958, and rs3211817, were also significantly associated with HDL-cholesterol (Table 3). The patterns were similar for log(triglycerides), triglycerides vs. HDL ratios, and the significance level was persistent after multiple comparison adjustments, indicating the *CD36* gene variants were significantly associated with triglycerides HDL-cholesterol, and systolic blood pressure. Figures 1, 2, 3 and 4 showed the impact of *CD36* gene variants on triglycerides, HDL-cholesterol and systolic blood pressure from pairwise genotype comparisons: subjects with rs3211848 homozygote had a higher triglycerides level (99.16 ± 2.61 mg/dL), compared with non-carriers (89.27 ± 1.45 mg/dL, *P* = 0.001). In addition, compared with non-carriers, individuals with rs1054516 heterozygous and homozygous genotypes had a significant lower HDL-cholesterol (46.6 ± 0.46 mg/dL for non-carrier, 44.6 ± 0.36 mg/dL for heterozygous, and 44.3 ± 0.56 mg/dL for homozygous, *P* = 0.0008 in codominant mode).

**Table 3:** **Association study for *CD36 *gene variants and metabolic syndrome components in the “normal” subjects, adjusted for age and gender, the numbers in bold shows the significant level (<0.05); Lipid Profiles & blood pressure**

**#rs**	**Log (Triglyceride)**				**Total cholesterol**			
	**Additive**	**Dominant**	**Recessive**	**Co-dominant**	**Additive**	**Dominant**	**Recessive**	**Co-dominant**
rs3211849	**0.013**	0.21	**0.001**	**0.005**	0.52	0.40	0.92	0.69
rs17154258	0.20	0.34	0.06	0.14	**0.029**	**0.022**	0.93	0.07
rs3211869	**0.026**	0.09	**0.034**	0.06	0.46	0.51	0.58	0.76
rs3211958	**0.019**	**0.040**	0.08	0.06	0.75	0.51	0.83	0.72
rs3211817	**0.019**	0.17	**0.007**	**0.021**	0.62	0.84	0.48	0.78
rs1054516	0.11	0.41	0.06	0.17	0.95	0.73	0.79	0.87
rs17154233	0.73	0.74	0.85	0.94	0.34	0.52	0.14	0.31
**#rs**	**HDL-C**				**SBP**			
	**Additive**	**Dominant**	**Recessive**	**Co-dominant**	**Additive**	**Dominant**	**Recessive**	**Co-dominant**
rs3211849	**0.002**	**0.002**	0.07	**0.006**	**0.002**	**0.013**	**0.011**	**0.009**
rs17154258	0.42	0.80	**0.004**	**0.017**	0.84	0.75	0.53	0.75
rs3211869	**0.001**	**0.016**	**0.001**	**0.002**	0.26	0.14	0.98	0.29
rs3211958	**0.001**	**0.001**	**0.035**	**0.002**	0.11	0.26	0.14	0.26
rs3211817	**0.003**	**0.027**	**0.007**	**0.010**	0.12	0.06	0.70	0.16
rs1054516	**0.001**	**0.000**	0.10	**0.001**	**0.002**	**0.004**	**0.025**	**0.007**
rs17154233	0.14	0.06	0.46	0.08	0.41	0.48	0.46	0.65
**#rs**	**log(TG/HDL-C)**				**DBP**			
	**Additive**	**Dominant**	**Recessive**	**Co-dominant**	**Additive**	**Dominant**	**Recessive**	**Co-dominant**
rs3211849	**0.002**	**0.033**	**0.001**	**0.002**	0.08	0.45	**0.017**	0.06
rs17154258	0.18	0.37	**0.013**	**0.041**	0.22	0.20	1.00	0.43
rs3211869	**0.003**	**0.026**	**0.004**	**0.006**	0.55	0.32	0.72	0.46
rs3211958	**0.002**	**0.003**	**0.032**	**0.007**	0.64	0.79	0.60	0.87
rs3211817	**0.003**	0.05	**0.002**	**0.004**	0.51	0.37	0.99	0.64
rs1054516	**0.011**	**0.042**	**0.033**	**0.039**	0.05	0.20	0.06	0.13
rs17154233	0.39	0.32	0.88	0.56	0.77	0.88	0.57	0.85

The clinical distribution of total participants, according to the SNP rs1054516 genotype status was listed in Additional file 1: Table S3. The distributions among three genotypes were similar, except for HDL cholesterol: compared with those with CC and TC genotype, participants with TT genotypes had a higher HDL cholesterol level (P = 0.009).

Table 4 showed the multiple regression models of SNP rs1054516 genotypes and various clinical variables, including age, gender, body mass index, metabolic syndrome status, smoking, drinking and sports activity, as well as the hypoglycemic and lipid lowering drugs. The estimates for SNP rs1054516, after controlling for multiple variables were significant: compared with TT genotype, TC and CC genotypes had lower HDL cholesterol levels (−1.00±0.40 mg/dL between TC and TT, P = 0.012, -1.19±0.49 mg/dL between CC and TT, P = 0.012). Besides, we found that age, gender, body mass index, metabolic syndrome status, drinking, and sports activity status were related to HDL cholesterol levels.

**Table 4:** The estimated parameters, standard errors and significant levels from the multiple linear regression models for HDL cholesterol levels in the study participants

		**Parameter Estimate**	**Standard error**	**P value**
Intercept		55.3	1.9	<.0001
SNP rs1054516	CC vs. TT	−1.19	0.49	0.015
	TC vs. TT	−1.00	0.40	0.012
age	+1 yr	0.05	0.02	0.008
Sex	Men vs. Women	−6.76	0.41	<.0001
BMI	+1 kg/m^2	−0.49	0.07	<.0001
Metabolic syndrome	case vs. control	−2.51	0.46	<.0001
Smoking	Yes vs. No	−0.51	0.51	0.31
Drinking habit	Yes vs. No	1.07	0.37	0.004
Sports activity	Frequent vs. No	1.45	0.39	0.0002
Hypoglycemic medication	Yes vs. No	−1.11	0.62	0.08
Lipid lowering medication	Yes vs. No	0.99	0.68	0.15

### Haplotype analysis frequencies and association study with metabolic syndrome components

We performed the haplotypes analyses for the 7 SNPs from the *CD36* gene to assess the association between these haplotypes and various continuous metabolic syndrome components. Table 5 lists the common haplotype (frequency > =5%) and the multivariate adjusted continuous traits, which showed significant reduction: individuals carrying the second common haplotype (GAAGGTA, frequency = 23.0%) had a significant level of HDL cholesterol (adjusted mean, 23.8, 95% confidence interval[CI], 22.3 to 25.4 mg/dL), as compared to those with the most frequent haplotype, AATATCA (26.7%, adjusted mean, 22.2, 95% CI, 20.7 to 23.6 mg/dL), *P* = 0.0001. The pattern was similar for triglycerides and triglycerides with regards to HDL-cholesterol ratio.

**Table 5:** **Common haplotypes (> = 5%) for *CD36 *gene associations with continuous metabolic factors among the study participants**

**Haplotypes**	**Frequency**	Multivariate adjusted *				**Mean**	**95% CI**		P**
		**Mean**	**95% CI**		P**				
		**HDL-cholesterol**				**log (TG vs. HDL)**			
AATATCA	0.267	22.2	20.7	23.6		0.312	0.236	0.388	
GAAGGTA	0.230	23.8	22.3	25.4	0.0001	0.246	0.164	0.328	0.006
GATATTA	0.098	22.3	20.4	24.1	0.86	0.330	0.234	0.426	0.60
GATGTTC	0.076	23.2	21.3	25.1	0.14	0.262	0.154	0.370	0.22
AATGTCA	0.058	22.3	20.3	24.4	0.82	0.317	0.205	0.429	0.91

The order of polymorphisms on the haplotype according to *CD36* SNPs.

*: adjusted for gender and age.

**: Compared with the most common haplotype.

## Discussion

Our study showed that the *CD36* gene variants were associated with triglycerides and HDL cholesterol concentrations among ethnic Chinese in Taiwan. We constructed the haplotypes of CD36 gene and extensively checked the association of specific SNP rs1054516 and other clinical variables with HDL cholesterol level. Our study has provided the following clinical evidence. First, the *CD36* candidate gene is an important determinant of HDL-cholesterol and triglycerides among individuals with normal-range lipids. Second, the metabolic syndrome itself, does not relate to the *CD36* gene polymorphism in our study.

The mechanism of the *CD36* gene for the pathogenesis of metabolic syndrome components has been proposed as the following: First, in the *CD36* knockout mice, defective uptake and utilization of long-chain fatty acids were found in muscle and adipose tissues [25]. Transgenic mice over-expression CD36 protein was found to have reduced blood lipids [26]. The transgenic expression of CD36 in the hypertensive rat model showed the decreased metabolic syndrome and insulin resistance burden. Cell culture study on monocyte CD36 expression showed a 34% expression increase in type 2 diabetes as compared with control subjects [27]. Second, in human cells, the CD36 protein receptor has a highly affinity to HDL; however, human LDL bound poorly to CD36 protein [28], indicating selective binding of CD36 protein to HDL, instead of to LDL. In addition, CD36 is a receptor for oxidized LDL and plays a role in scavenging LDL modified by oxidation and mediating the atherosclerosis process [1]. The expression patterns of *CD36* gene in human tissues were up-regulated in response to oxidized LDL [29]. Furthermore, CD36 protein facilitates a large fraction of fatty acid uptake and CD36 deficiency in humans was reported defect in myocardial cell fatty acid analog [25], and this effect on cardiac triglycerides storage was enhanced in obese status [30]. In CD36-null mice, diet high in fructose induced markedly glucose intolerance and hyperinsulinemia [31]. In addition, consuming saturated fatty acid diets would increase LDL cholesterol level in the scavenger receptor class B type 1 gene variant, a similar effect as *CD36* gene [32]. Our data supported *CD36* genetic effects on triglycerides and HDL cholesterol.

### Evidence for *CD36* gene in dyslipidemia

Previous genetic studies showed various effects between *CD36* locus and metabolic syndrome components. A genome-wide linkage scan among 418 individuals from 27 extended Mexican American families found that two loci in chromosome 7 were suggested as being linked to HDL cholesterol and triglycerides levels, besides the most linkage site in chromosome 15q [33]. In addition, among non-diabetic Mexican American families, quantitative trait locus study showed a strong linkage of one metabolic syndrome related factor, HDL and triglycerides, to chromosome 7 (LOD score up to 3.2) [34]; however, other metabolic factors, including obesity and blood pressure, cannot be identified to linkage to chromosome 7, in which *CD36* gene exists. Moreover, another large-scale genome-wide linkage scan among 8664 participants from multiple ethnicities showed that 7q36, a site for *CD36*, was associated with fasting glucose and insulin resistance [35]. To sum up, a meta-analysis based on genome-wide linkage studies on quantitative lipid traits from the families ascertained from type 2 diabetes showed that *CD36* gene locus (7p11-q21.11) was significantly linked to triglycerides and triglycerides/HDL cholesterol ratio, but not linked to LDL or total cholesterol [36]. Our findings also demonstrated significant association between *CD36* gene polymorphism and triglycerides and HDL cholesterol, the traits predisposing to type 2 diabetes. In another study based on 1,375 patients with coronary heart disease, one SNP in *CD36*, rs3211956, was significantly associated with acute myocardial infarction as compared with stable coronary disease (allele frequency 11% vs. 8%, p = 0.04) [6]. However, the strength decreased modestly after adjusting covariates and multiple comparisons. Among a cohort composed of 675 obese adults (age >40 yrs and body mass index >25) in the Netherlands, rs1527479, a C/T SNP in the upstream promoter region in the *CD36* gene, homozygous carrier was associated with prevalent type 2 diabetes, and more so in women and high BMI (>27) group [8]. Furthermore, the homozygous carriers were more likely to have a high homeostasis model assessment index value [8]. Another case–control study on 61 *CD36*-deficient patients and 25 controls showed that the patients were likely to have a higher type 2 diabetes prevalence, fasting glucose, glycated hemoglobin and HDL-cholesterol, and likely to have a lower triglyceride value [4]. In addition, the study based on screening the coding sequence of the *CD36* gene in 272 French individuals showed that one promoter variant allele (−178A/C) was associated with adiponectin levels (*p* = 0.036 after multiple testing correction) [11]. Among another French family study, a rare nonsense mutation (1079 T > G) in *CD36* locus showed linkage with familial type 2 diabetes risk [7]. In addition, genotyping 21 SNPs in the *CD36* gene in 585 non-diabetic Caucasians, Ma and colleagues showed that 5 tagged SNPs for haplotype construction [9]. The 30294 G > C polymorphism was associated with free fatty acid level (p = 0.02) and the association was apparent among men. Compared with non-carriers, individuals carrying the haplotype AGGIG had a 31% higher free fatty acids (p = 0.0002) and 20% higher triglycerides (p = 0.025) [9]. Furthermore, this haplotype was associated with a higher risk of coronary heart disease among type 2 diabetic patients. However, a survey based on 831 adults from the health screening showed that one *CD36* gene variant (Pro90Ser) was associated with free fatty acids, but not related to HDL cholesterol nor triglycerides [12]. Extensive tagged SNP study on *CD36* gene among African-Americans showed this gene was associated with metabolic syndrome and HDL cholesterol [10]. A genome wide association study base on more than ten thousand individuals showed that biological lipoprotein metabolism related genes, such as *CYP7A1, NPC1L1* and *SCARB1*, were related to lipid profiles [37]. Our study showed that the *CD36* variants with differential effects on triglycerides and HDL cholesterol, consistent with previous findings.

Our study has several strengths. First, the study sample size was moderate, which provided us with sufficient statistical power. In addition, the selection strategy on common htSNPs from public domain (HapMap website) could reduce genotyping costs, and provides an important tool to explore the candidate gene effects. Second, this study population is relatively homogenous and thus may reduce the effect of population stratification. We recruited the participants from the health checkup center in a tertiary university hospital, and these participants had relatively high socioeconomic status and their health behavior and health promotion motivation were high. Third, due to the high prevalence of dyslipidemia and high blood pressure in the control subjects, we believe the heterogeneity in the metabolic syndrome reduced the association strength between gene and the metabolic syndrome. Further study on the pathogenesis of *CD36* gene expression and metabolic components, especially triglycerides and HDL cholesterol levels, is warranted.

This study has some limitations. First, some subjects of the metabolic syndrome may be due to phenocopies and other environmental factors, rather than genetic effects, causing the traits to be the same. This was apparently due to the relatively middle and elderly participants in our study. These phenocopies and misclassification would reduce the power of the association and it may bias our results toward the null. Second, although our study had sufficient statistical power to detect large effects resulting from common alleles, the power to evaluate small effects due to rare alleles or the effect of interaction was limited. Further gene-environment interaction study needs greater study numbers. Studying complex diseases has been shown to require very large sample sizes as multiple small effects may be expected. The study population in this study does exceed many very small studies, but may still lack power to detect small associations. Third, we did not include other genetic information, such as *APOE, APOA5,* and *LPL* genes. Indeed, our previous studies have shown that the *APOA5* and *APOA1-C3-A4* genetic polymorphism was associated with dyslipidemia in Taiwanese population [15,38-40]. Finally, due to different components of the metabolic syndrome, multiple comparison issues may be a concern for inflating type I error.

In conclusion, our genetic association study demonstrated that *CD36* gene variants were significantly associated with triglycerides and HDL-cholesterol levels. Further study on the pathogenesis of *CD36* gene expression and metabolic components, especially with regards to triglycerides and HDL cholesterol levels, is warranted.

## Competing interests

The authors declare that they have no competing interests.

## Authors’ contributions

KLC, HCH and MFC proposed the study design. KLC, HJL, and BCL participated in data collection. KLC and PHL performed statistical and genetic analysis. HCH performed lipids and laboratory measurements and quality control. KLC and MFC conceived of the study, and participated in its design and coordination and helped to draft the manuscript. All authors read and approved the final manuscript.

## Supplementary Material

Additional file 1: Table S1 Minor Allele Frequency of the selected SNPs of CD36 gene in the study participants. **Table S2.** Selected SNPs for significant association between genetic polymorphisms and metabolic syndrome status as well as components in the study participants (200/200 cases-controls), according to adjusted status (age, gender-adjusted) and the modes of inheritance, including the co-dominant, additive, dominant and recessive models. **Table S3.** Clinical characteristics of the study participants according to SNP rs1054516 genotype status. Click here for file
