# Supplementary material for: Common sequence variants in CD36 gene and the levels of triglyceride and high-density lipoprotein cholesterol among ethnic Chinese in Taiwan
Source: Lipids Health Dis. 2012 Dec 18;11:174. doi: 10.1186/1476-511X-11-174 (PMC3575328; doi:10.1186/1476-511X-11-174)
Supplement: Additional file 1: Table S1 — Minor Allele Frequency of the selected SNPs of CD36 gene in the study participants. Table S2. Selected SNPs for significant association between genetic polymorphisms and metabolic syndrome status as well as components in the study participants (200/200 cases-controls), according to adjusted status (age, gender-adjusted) and the modes of inheritance, including the co-dominant, additive, dominant and recessive models. Table S3. Clinical characteristics of the study participants according to SNP rs1054516 genotype status. [file 1476-511X-11-174-S1.doc]

**Supplementary Table 1: Minor Allele Frequency of the selected SNPs of *CD36* gene in the study participants,**

| rs number | Assay ID | Han Chinese (HCB) |
| --- | --- | --- |
| rs3211850 | [C__32339926_10](javascript:showAssayDetails('https://products.appliedbiosystems.com:443/ab/en/US/adirect/ab?cmd=ABAssayDetailDisplay&assayID=C__32339926_10&Fs=y');) | 0.0 (A) |
| rs7755 | [C___8315318_10](javascript:showAssayDetails('https://products.appliedbiosystems.com:443/ab/en/US/adirect/ab?cmd=ABAssayDetailDisplay&assayID=C___8315318_10&Fs=y');) | 0.47 (A) |
| rs3211845 | [C__32339930_10](javascript:showAssayDetails('https://products.appliedbiosystems.com:443/ab/en/US/adirect/ab?cmd=ABAssayDetailDisplay&assayID=C__32339930_10&Fs=y');) | 0.1 (A) |
| rs3211867 | [C___1803793_10](javascript:showAssayDetails('https://products.appliedbiosystems.com:443/ab/en/US/adirect/ab?cmd=ABAssayDetailDisplay&assayID=C___1803793_10&Fs=y');) | 0.25 (A) |
| rs3211958 | [C__11847639_10](javascript:showAssayDetails('https://products.appliedbiosystems.com:443/ab/en/US/adirect/ab?cmd=ABAssayDetailDisplay&assayID=C__11847639_10&Fs=y');) | 0.39 (G) |
| rs3173800 | [C___1803779_20](javascript:showAssayDetails('https://products.appliedbiosystems.com:443/ab/en/US/adirect/ab?cmd=ABAssayDetailDisplay&assayID=C___1803779_20&Fs=y');) | 0.26 (T) |
| rs3211820 | [C__29171553_20](javascript:showAssayDetails('https://products.appliedbiosystems.com:443/ab/en/US/adirect/ab?cmd=ABAssayDetailDisplay&assayID=C__29171553_20&Fs=y');) | - |
| rs1054516 | [C__12101678_10](javascript:showAssayDetails('https://products.appliedbiosystems.com:443/ab/en/US/adirect/ab?cmd=ABAssayDetailDisplay&assayID=C__12101678_10&Fs=y');) | 0.5 (C) |
| rs3211879 | [C___1803784_10](javascript:showAssayDetails('https://products.appliedbiosystems.com:443/ab/en/US/adirect/ab?cmd=ABAssayDetailDisplay&assayID=C___1803784_10&Fs=y');) | 0.02 (A) |
| rs3211869 | [C__31374630_10](javascript:showAssayDetails('https://products.appliedbiosystems.com:443/ab/en/US/adirect/ab?cmd=ABAssayDetailDisplay&assayID=C__31374630_10&Fs=y');) | 0.21 (A) |
| rs3211956 | [C__27519229_10](javascript:showAssayDetails('https://products.appliedbiosystems.com:443/ab/en/US/adirect/ab?cmd=ABAssayDetailDisplay&assayID=C__27519229_10&Fs=y');) | 0.21 (G) |
| rs17154232 | [C__33463947_10](javascript:showAssayDetails('https://products.appliedbiosystems.com:443/ab/en/US/adirect/ab?cmd=ABAssayDetailDisplay&assayID=C__33463947_10&Fs=y');) | 0.07 (C) |
| rs3211883 | [C___1803782_20](javascript:showAssayDetails('https://products.appliedbiosystems.com:443/ab/en/US/adirect/ab?cmd=ABAssayDetailDisplay&assayID=C___1803782_20&Fs=y');) | 0.31 (A) |
| rs3212008 | [C__32339901_10](javascript:showAssayDetails('https://products.appliedbiosystems.com:443/ab/en/US/adirect/ab?cmd=ABAssayDetailDisplay&assayID=C__32339901_10&Fs=y');) | 0.0 (T) |
| rs3211863 | [C__32339917_10](javascript:showAssayDetails('https://products.appliedbiosystems.com:443/ab/en/US/adirect/ab?cmd=ABAssayDetailDisplay&assayID=C__32339917_10&Fs=y');) | 0.01 (G) |
| rs17154233 | [C__33463946_10](javascript:showAssayDetails('https://products.appliedbiosystems.com:443/ab/en/US/adirect/ab?cmd=ABAssayDetailDisplay&assayID=C__33463946_10&Fs=y');) | 0.13 (C) |
| rs3211908 | [C__31374621_10](javascript:showAssayDetails('https://products.appliedbiosystems.com:443/ab/en/US/adirect/ab?cmd=ABAssayDetailDisplay&assayID=C__31374621_10&Fs=y');) | 0.16 (T) |

**Supplementary Table 2: Selected SNPs for significant association between genetic polymorphisms and metabolic syndrome status as well as components in the study participants (200/200 cases-controls), according to adjusted status (age, gender-adjusted) and the modes of inheritance, including the co-dominant, additive, dominant and recessive models**

| **Rs#** | **Codo** | **Add** | **Dom** | **Rec** | **traits** |
| --- | --- | --- | --- | --- | --- |
| rs17154232 |  |  |  |  | logtghdl |
| rs3211931 | ***** | ***** | ***** |  | logac |
| rs3211958 |  |  |  |  | logtg |
| rs3211958 | ***** |  | ****** |  | logac |
| rs3211817 |  |  | ***** |  | bmi |
| rs3211817 | ***** | ****** | ****** |  | hdl |
| rs17154233 |  |  |  | ***** | wc |
| rs17154233 | ***** | ***** | ***** |  | logtg |
| rs3211883 |  |  |  |  | hdl |
| rs3211883 |  | ***** |  |  | logac |
| rs3211879 |  |  | ***** |  | logac |
| rs3211869 | ***** | ****** | ****** |  | hdl |
| rs3211908 |  |  | ***** |  | logac |
| rs1054516 |  |  |  | ***** | logtg |
| rs1054516 | ****** | ****** | ****** |  | hdl |
| rs3173798 | ***** | ***** |  | ***** | logac |
| rs17154258 |  | ***** | ***** |  | bmi |
| rs17154258 | ***** | ***** | ***** |  | wc |
| rs17154258 | ****** |  |  | ****** | hdl |
| rs17154258 |  |  |  |  | logtghdl |
| rs3211886 |  | ***** |  |  | logtg |
| rs3211816 |  |  |  | ***** | logtg |
| rs3211816 |  |  |  | ***** | logtghdl |
| rs3211849 | ***** |  |  |  | mets |
| rs3211849 |  | ***** | ***** |  | hdl |
| rs3211849 |  | ***** |  |  | logac |

Mode of inheritance: Codo, co-dominant; Add, additive; Dom, dominant; Rec, recessive;

blank as >0.05, * as 0.05 ~ 0.01, ** as 0.01 ~ 0.001, *** as 0.001~0.0001

**Supplementary Table 3: Clinical characteristics of the study participants according to SNP rs1054516 genotype status**

|  | CC |  | TC |  | TT |  |  |
| --- | --- | --- | --- | --- | --- | --- | --- |
|  | N=392 |  | N=927 |  | N=577 |  | P |
|  | N | % | N | % | N | % |  |
| Gender |  |  |  |  |  |  |  |
| Women | 144 | 36.7 | 345 | 37.2 | 217 | 37.6 | 0.96 |
| Men | 248 | 63.3 | 582 | 62.8 | 360 | 62.4 |  |
| Hypoglycemic drugs (Yes) | 32 | 8.2 | 100 | 10.8 | 45 | 7.8 | 0.10 |
| Lipid lowering drugs (yes) | 25 | 6.4 | 72 | 7.8 | 45 | 7.8 | 0.64 |
| Metabolic syndrome |  |  |  |  |  |  | 0.46 |
| Control | 203 | 51.8 | 446 | 48.1 | 287 | 49.7 |  |
| Case | 189 | 48.2 | 481 | 51.9 | 290 | 50.3 |  |
| Current smoker | 66 | 16.8 | 156 | 16.8 | 82 | 14.2 | 0.36 |
| Current drinker | 228 | 58.2 | 525 | 56.6 | 328 | 56.9 | 0.87 |
| Regular sports habit | 273 | 69.6 | 625 | 67.4 | 403 | 69.8 | 0.55 |
|  | Mean | SD | Mean | SD | Mean | SD |  |
| Age, yrs | 53.9 | 10.7 | 54.9 | 10.5 | 55.3 | 10.7 | 0.16 |
| Body mass index, kg/m^2^ | 25.0 | 3.4 | 25.1 | 3.5 | 24.8 | 3.5 | 0.27 |
| Waist circumference, cm | 86.4 | 9.8 | 87.1 | 10.2 | 86.6 | 10.2 | 0.45 |
| Systolic blood pressure, mmHg | 126.7 | 16.2 | 128.2 | 16.1 | 128.5 | 14.8 | 0.19 |
| Diastolic blood pressure, mmHg | 75.7 | 11.1 | 76.2 | 10.6 | 76.0 | 9.9 | 0.69 |
| Total cholesterol, mg/dL | 205.0 | 36.1 | 204.7 | 36.2 | 205.3 | 37.6 | 0.95 |
| Triglycerides, mg/dL | 144.9 | 90.5 | 148.3 | 99.9 | 145.1 | 92.3 | 0.75 |
| HDL cholesterol, mg/dL | 40.4 | 8.3 | 40.4 | 8.3 | 41.8 | 9.7 | 0.009 |
| LDL cholesterol, mg/dL | 121.6 | 30.9 | 120.5 | 32.4 | 120.2 | 33.5 | 0.80 |
